# Distribution of Corneal Geometric Landmarks and Relationship Between Their Distances and Biomechanical Parameters in the Development of Keratoconus

**DOI:** 10.3389/fbioe.2021.766163

**Published:** 2021-12-22

**Authors:** Lei Tian, Hui Zhang, Li-Li Guo, Xiao Qin, Di Zhang, Lin Li, Ying Wu, Ying Jie, Haixia Zhang

**Affiliations:** ^1^ Beijing Institute of Ophthalmology, Beijing Tongren Eye Center, Beijing Tongren Hospital, Capital Medical University and Beijing Ophthalmology and Visual Sciences Key Laboratory, Beijing, China; ^2^ Beijing Advanced Innovation Center for Big Data-Based Precision Medicine, Beihang University & Capital Medical University, Beijing, China; ^3^ School of Biomedical Engineering, Capital Medical University, Beijing, China; ^4^ Department of Medical Engineering, Peking Union Medical College Hospital, Chinese Academy of Medical Sciences, Beijing, China; ^5^ The First People's Hospital of Xuzhou, Jiangsu, China; ^6^ Beijing Key Laboratory of Fundamental Research on Biomechanics in Clinical Application, Capital Medical University, Beijing, China; ^7^ Department of Ophthalmology, Chinese People's Liberation Army General Hospital, Beijing, China

**Keywords:** keratoconus, forme fruste keratoconus, morphology, biomechanics, geometric landmark

## Abstract

**Purpose:** To analyze the changes in coordinates and distances among three typical geometric landmarks of the cornea, namely, the thinnest point (TP), maximum curvature (Kmax), and corneal apex (AP) during the development of keratoconus, and explore the potential relationship between these changes and the abnormalities of corneal biomechanics.

**Methods:** Normal eyes (*n* = 127), clinical keratoconic eyes (CKC, *n* = 290), and the eyes of forme fruste keratoconus (FFKC, *n* = 85) were included; among them, the CKC group was classified into four grades based on the Topographic Keratoconus Classification (TKC) provided by Pentacam. A total of 38 Corvis ST output parameters and three distance parameters of three typical landmarks (D_Kmax-AP_, D_TP-AP_, and D_Kmax-TP_) based on Pentacam were included. The differences of parameters among the abovementioned six groups (Normal, FFKC, and CKC stage I to CKC stage IV) were analyzed. Spearman’s rank correlation test was performed to choose several dynamic corneal response (DCR) parameters that could best reflect the changes of corneal biomechanical characteristics during the progression of the disease, and the Pearson’s or Spearman’s correlation test was conducted to determine the association between the three distances and the selected DCR parameters in each grade. In addition, by flipping the X coordinate of the left eye on the vertical axis to reflect the direction of the right eye, the coordinates of TP and Kmax in different developmental grades were highlighted.

**Results:** From CKC stage II, the three geometric landmark distances commenced to correlate with the corneal DCR parameters (CBI, SPA1, IR, DA Ratio 2, ARTh, MIR, Radius, Pachy, and DA Ratio 1), which could better represent the changes of biomechanical properties from normal cornea to keratoconus. From normal cornea to CKC stage IV, the coordinates of Kmax were gradually tended to the inferior temporal region from dispersion, while TP was always concentrated in the inferior temporal region. Although D_Kmax-AP_, D_Kmax-TP_, and D_TP-AP_ all showed a gradual decreasing trend with the progress of the disease, the first two did not change significantly, and only D_TP-AP_ significantly approached AP in the later stage of disease development. In addition, from the FFKC group, the corresponding values of D_Kmax-TP_ in each disease development group were smaller than D_Kmax-AP_.

**Conclusions:** In the later stage of keratoconus, the relationship between the three typical landmark distance parameters and DCR parameters is stronger, and even the weakening of corneal biomechanical properties may be accompanied by the merger of typical landmark positions.

## Introduction

With respect to in-depth understanding of the biomechanical mechanism of corneal diseases, evaluation of the biomechanical properties of cornea has greatly attracted scholars’ attention in terms of prevention and treatment of corneal dilatation diseases, especially keratoconus (Herber et al., 2019). It has been gradually found that the biomechanical properties of the cornea depend on the collagen fiber, collagen fiber bundle, and their spatial structure composition (Oxlund and Simonsen, 1985), and studies (Meek et al., 2005; Catalán-López et al., 2018) have shown that the mechanical strength of keratoconus is often remarkably weaker than that of normal cornea. In addition, a number of scholars have pointed out that the morphological changes of keratoconus may be secondary to the changes in corneal biomechanics (Roberts et al., 2017; Sedaghat et al., 2018).

At present, the Ocular Response Analyzer (ORA) and Corneal Visualization Scheimpflug Technology (Corvis ST) are the two most recognized devices for the measurement of cornea biomechanics *in vivo*. Among them, ORA cannot display the process of corneal deformation dynamically in real time, and its main biomechanical parameters, corneal hysteresis (CH) and corneal resistance factor (CRF), are derived by analyzing the measured waveforms (McMonnies, 2012), while Corvis ST can dynamically record the whole process under impulse pressure and generate DCR parameters to reflect the biomechanical characteristics of cornea (Elham et al., 2017). For now, the combined diagnosis of keratoconus with Corvis ST and three-dimensional anterior segment analysis and diagnosis system Pentacam, which characterizes the morphological features of cornea, has been clinically recognized.

That is to say, although morphological changes are not the primary cause of keratoconus, its diagnostic value cannot be easily ignored. As we all know, the most typical morphological change of keratoconus is the thinning and protruding of cornea (Hashemi et al., 2019). To date, numerous studies (Galletti et al., 2015; Chan et al., 2018) have concentrated on the analysis of the numerical changes of the thinnest thickness and the maximum curvature caused by the gradual corneal protrusion, while few people have analyzed the coordinates of these two typical geometric landmarks and the distance between them and the central apex of the cornea. Only several studies simply compared the distance between the cornea apex and the thinnest point of cornea in different groups, and found that there were remarkable differences among normal cornea, subclinical keratoconus (Muftuoglu et al., 2013), and clinical keratoconus (Muftuoglu et al., 2015).

As mentioned above, DCR parameters and distance parameters of typical geometric landmarks representing morphological characteristics are both significantly different between normal cornea and keratoconus. Thus, we speculated that there may be a certain correlation between the DCR parameters and distance parameters with the development of keratoconus disease.

The main purpose of the present study was to evaluate the relationship between the distances among the typical geometric landmarks of cornea and the DCR parameters output by Corvis ST in the assumed different grades of keratoconus development, and explore more potential patterns of disease development.

## Patients and Methods

### Study Subjects

This prospective comparative study included patients with clinical keratoconus (CKC group), forme fruste keratoconus (FFKC group), and candidates undergoing refractive surgery with normal corneas (Normal group).

A diagnosis of keratoconus was made if the eye met the following conditions (Tian et al., 2014; Cui et al., 2016): (1) an irregular cornea determined by distorted keratometry mires, distortion of the retinoscopic or ophthalmoscopic red reflex (or a combination of the two); (2) with at least one of the following biomicroscopic signs: Vogt’s striae, Fleischer’s ring of >2 mm arc, or corneal scarring consistent with keratoconus. In the CKC group, the seven classes of topographic keratoconus classification (TKC) provided by Pentacam (Oculus, Wetzlar, Germany) were included (i.e., 1, 1-2, 2, 2-3, 3, 3-4, and 4). Those classes could be divided into four four subgroups based on their TKC number: TKC 1 was classified as CKC stage I, TKC 1-2 and 2 were classified as CKC stage II, TKC 2-3 and 3 were classified as CKC stage III, and TKC 3-4 and 4 were classified as CKC stage IV.

An eye was diagnosed with FFKC if it was the fellow eye of a patient with keratoconus and showed the following features (Peña-García et al., 2016): (1) a normal-appearing cornea on slit-lamp examination, retinoscopy, and ophthalmoscopy; (2) topography was normal with no asymmetric bowtie and no focal or inferior steepening pattern; (3) the level of TKC was normal, namely, it was “-”; and (4) the patient had no history of contact lens use, ocular surgery, or trauma. For participants undergoing refractive surgery, only one eye from each individual was chosen using a random numbers table. In addition, the TKC level of eyes in the Normal group and FFKC group was normal.

Exclusion criteria were a history of undergoing ocular surgery and cases with eye diseases that may potentially affect the outcomes. For contact lens-wearing patients, they were asked to remove soft contact lenses at least 2 weeks and rigid contact lenses at least 1 month before assessment. Data were collected from May 2013 to January 2020 at the Beijing Tongren Hospital affiliated to Capital Medical University (Beijing, China). All participants signed the written informed consent form prior to commencing the study. The study was carried out in accordance with the Declaration of Helsinki, and it was approved by the Ethics Committee of the Beijing Tongren Hospital affiliated to Capital Medical University.

### Ocular Examination

A comprehensive ocular examination was performed on all eyes, including uncorrected visual acuity, slit-lamp and fundoscopic examinations, Pentacam tomographic examination, and Corvis ST (Oculus; Wetzlar, Germany) measurements. All measurements were undertaken between 9:00 a.m. and 5:00 p.m. by the same trained ophthalmologists during the same visit.

### Pentacam Measurement

The Pentacam software (ver. 1.20r134) reconstructs a three-dimensional (3D) image of the entire anterior segment from the anterior surface of the cornea to the posterior surface of the lens by utilizing the high-speed rotating Scheimpflug system. Details and principles of the Pentacam are described elsewhere (Cui et al., 2016). Only scans that the Pentacam’s “quality specification” (QS) function determined as “OK” were included for analysis.

In the study, we focused on three points on the cornea: corneal apex (AP), thinnest point (TP), and maximum curvature (Kmax). Extracted parameters from Pentacam data for analysis included the coordinates of TP (TP X, TP Y) and Kmax (Kmax X, Kmax Y); then, we calculated the absolute distances from the cornea apex (geometric center of the examination [x = 0; y = 0]): D_TP-AP_ and D_Kmax-AP_, and the distance between Kmax and TP (D_Kmax-TP_). The formula of distance was as follows:

$$\text{d}=\;\sqrt{{\left(x_{1}-x_{2}\right)}^{2}+{\left(y_{1}-y_{2}\right)}^{2}\;}$$

### Corvis ST Measurement

The Corvis ST software (ver. 1.5r1902) measures dynamic corneal deformation response to an air-puff pulse. Details and principles of the Corvis ST are described elsewhere (Elham et al., 2017). The measurements were checked under the QS window; only correct measurements were accepted (comment box reading “OK”). If the comment box was marked yellow or red, the examination was repeated. The following parameters were detected by Corvis ST: intraocular pressure (IOP), Pachymetry (Pachy), time from the initiation of air puff until the first applanation (A1T), second applanation (A2T) and maximum deformation (HCT), corneal velocity at the first (A1V) and second applanation (A2V), peak distance (PD) and radius of curvature (Radius), deformation amplitude at the first applanation (A1DA), second applanation (A2DA) and highest concavity (HCDA), deflection length at the first applanation (A1DLL), second applanation (A2DLL) and highest concavity (HCDLL), deflection amplitude at the first applanation (A1DLA), second applanation (A2DLA) and highest concavity (HCDLA), deflection area at the first applanation (A1DLAr), second applanation (A2DLAr) and highest concavity (HCDLAr), delta arc length at the first applanation (A1dArcL), second applanation (A2dArcL) and highest concavity (HCdArcL), max time and length at deflection amplitude (DLAMT, DLAML), max time and amplitude of whole eye movement (WEMT, WEMA), delta arc length max (dArcLM) and PachySlope, the maximal value of the ratio between deformation amplitude at the apex and that at 1 (DA Ratio 1) and 2 mm (DA Ratio 2) from the corneal apex, max inverse radius (MIR) and integrated radius (IR), Ambrósio relational thickness to the horizontal profile (ARTh), Biomechanical-corrected intraocular pressure (bIOP), stiffness parameter at first applanation (SPA1), and Corvis biomechanical index (CBI).

### Statistical Analysis

Statistical analysis was performed using SPSS 20.0 (IBM, Armonk, NY, USA). Drawing was completed by R Core Team (version 3.6.1; R Foundation for Statistical Computing, Vienna, Austria; https://www.R-project.org/) software and GraphPad Prism software version 8.0, respectively.

The Shapiro–Wilk test was used to assess normal distribution of quantitative data. The normally distributed data were expressed as the mean ± standard deviation (SD), while abnormally distributed data were presented as median and range of variation.

One-way analysis of variance (ANOVA) test or non-parametric Kruskal–Wallis test was used to analyze the differences among the four subgroups of CKC group, Normal group, and FFKC group. Spearman’s rank correlation test was performed to assess correlation among all parameters measured by Corvis ST and the developmental grades of keratoconus (Rank-group). Then, the Pearson’s or Spearman’s correlation test was applied to determine the association between the distance of three geometric landmarks and the above selected DCR parameters in each grade, and Bonferroni correction was performed (*p* < 0.0056). Moreover, we plotted the variation trend of three typical landmark distance parameters and the selected DCR parameters with the progress of disease stage. The differences of parameters in any disease stage and its adjacent previous disease stage were compared by independent sample *t*-test or nonparametric Mann–Whitney test, and Bonferroni correction was performed (*p* < 0.01).

In addition, with flipping the X coordinate of the left eye on the vertical axis to reflect the direction of the right eye, the coordinates of Kmax and TP in different developmental grades of keratoconus were drawn. *p* < 0.05 was considered statistically significant, except for the Bonferroni correction.

## Results

Herein, 290 eyes of 223 patients (mean age, 23.19 ± 7.39 years old; range of age, 9–53 years old) were assigned to the CKC group, of whom both eyes of 59 patients were included, one eye of 85 patients was involved because of unilateral keratoconus, and one eye of 79 patients was included because the fellow eye had undergone eye surgery or the quality of the examination did not meet the predefined criteria. The normal contralateral eye of the unilateral keratoconus constituted the FFKC group (mean age, 23.61 ± 7.73 years old; range of age, 10–49 years old). The Normal group consisted of 127 normal individuals (mean age, 24.39 ± 4.38 years old; range of age, 15–37 years old), and only one eye per person was randomly evaluated. There were no statistically significant differences between the groups in age distribution (*p* = 0.065, the Kruskal–Wallis test).

With the exception of D_Kmax-AP_, HCT, A2DA, A2DLL, DLAMT, and WEMA, statistically significant differences in other parameters were found among four subgroups of the CKC group, Normal group, and FFKC group (*p* < 0.05) (Table 1).

**TABLE 1:** Comparison of distance of three geometric landmarks and DCR parameters by groups.

	Normal group (*n* = 142)	FFKC group (*n* = 93)	CKC group (*n* = 290)				*p*-value
			Stage I (*n* = 54)	Stage II (*n* = 123)	Stage III (*n* = 82)	Stage IV (*n* = 31)	
Geometric landmarks distances							
D_Kmax-AP_ [mm]	1.02 (0.06–4.78)	1.13 (0.06–5.13)	1.12 (0.13–3.03)	1.17 ± 0.66	0.89 (0.13–2.84)	0.85 ± 0.59	0.058^a^
D_TP-AP_ [mm]	0.72 ± 0.19	0.76 (0.29–1.92)	0.77 ± 0.30	0.74 (0.06–2.96)	0.58 (0.13–2.25)	0.41 (0.18–1.26)	<0.001^a^
D_Kmax-TP_ [mm]	1.04 (0.01–5.17)	1.16 (0.14–5.00)	0.98 (0.21–2.52)	0.93 (0.08–2.45)	0.83 ± 0.40	0.43 (0.13–1.91)	<0.001^a^
DCR parameters (Corvis ST’s output parameters)							
IOP [mmHg]	14.5 (9.0–30.5)	13.6 ± 2.1	13.5 (8.0–30.0)	11.5 (5.0–19.0)	10.5 ± 2.3	10.4 ± 3.0	<0.001^a^
Pachy [μm]	543 ± 34	518 ± 33	502 ± 26	479 (402–591)	461 ± 32	449 ± 37	<0.001^a^
A1T [ms]	7.319 (6.782–8.990)	7.172 ± 0.225	7.156 (6.590–8.934)	6.992 ± 0.240	6.857 ± 0.222	6.873 ± 0.287	<0.001^a^
A1V [m/s]	0.146 ± 0.021	0.158 (0.095–0.196)	0.160 ± 0.021	0.170 ± 0.020	0.181 ± 0.023	0.185 ± 0.020	<0.001^a^
A2T [ms]	21.892 ± 0.400	21.961 ± 0.378	21.902 ± 0.378	22.132 ± 0.350	22.249 ± 0.338	22.260 ± 0.396	<0.001^b^
A2V [m/s]	−0.277 (−0.363 to −0.118)	−0.288 (−0.356 to −0.166)	−0.297 ± 0.044	−0.322 ± 0.046	−0.345 (−0.562 to −0.149)	−0.348 ± 0.049	<0.001^a^
HCT [ms]	16.863 (15.477–18.108)	16.863 (15.246–18.249)	17.094 (15.708–17.787)	16.863 (15,477–18.018)	17.094 (14.784–17.787)	16.772 ± 0.409	0.098^a^
PD [mm]	5.194 (4.228–5.768)	5.209 ± 0.268	5.142 ± 0.289	5.268 ± 0.286	5.311 ± 0.278	5.283 (4.569–5.559)	0.009^a^
Radius [mm]	7.387 ± 0.766	6.833 ± 0.768	6.316 ± 0.678	5.850 (4.152–9.040)	5.391 ± 0.670	5.090 ± 0.867	<0.001^a^
A1DA [mm]	0.131 ± 0.011	0.132 (0.098–0.150)	0.136 ± 0.010	0.137 (0.105–0.191)	0.141 ± 0.015	0.154 ± 0.017	<0.001^a^
HCDA [mm]	1.074 ± 0.110	1.116 ± 0.110	1.138 ± 0.116	1.192 (0.943–1.676)	1.281 (1.037–1.877)	1.298 (1.125–1.756)	<0.001^a^
A2DA [mm]	0.373 (0.244–0.617)	0.373 ± 0.068	0.381 ± 0.064	0.367 (0.226–0.567)	0.398 ± 0.074	0.394 (0.268–0.702)	0.246^a^
A1DLL [mm]	2.313 (1.835–2.895)	2.300 (1.891–2.684)	2.319 ± 0.129	2.339 ± 0.143	2.362 ± 0.184	2.394 ± 0.173	0.008^a^
HCDLL [mm]	6.832 (5.614–7.788)	6.708 ± 0.498	6.575 ± 0.518	6.674 ± 0.460	6.610 ± 0.472	6.380 ± 0.496	0.003^a^
A2DLL [mm]	2.716 (1.660–4.234)	2.652 (1.630–4.510)	2.608 (1.582–6.441)	2.866 ± 0.642	2.910 ± 0.680	2.877 ± 0.510	0.910^a^
A1DLA [mm]	0.094 ± 0.008	0.095 ± 0.007	0.099 ± 0.008	0.103 (0.079–0.166)	0.110 ± 0.014	0.120 ± 0.016	<0.001^a^
HCDLA [mm]	0.923 ± 0.104	0.965 ± 0.105	0.967 ± 0.116	1.061 ± 0.134	1.122 (0.870–1.734)	1.158 ± 0.122	<0.001^a^
A2DLA [mm]	0.103 (0.080–0.142)	0.107 (0.080–0.142)	0.104 (0.078–0.162)	0.115 (0.090–0.250)	0.124 (0.083–0.209)	0.138 ± 0.017	<0.001^a^
DLAML [mm]	0.938 ± 0.105	0.979 ± 0.111	0.972 ± 0.131	1.063 (0.825–1.558)	1.140 (0.905–1.936)	1.169 ± 0.120	<0.001^a^
DLAMT [ms]	16.388 (14.637–17.455)	16.334 (13.677–17.684)	16.436 (14.350–17.396)	16.438 (14.535–17.605)	16.297 (8.419–17.397)	16.219 ± 0.609	0.978^a^
WEMA [mm]	0.270 (0.156–0.504)	0.271 ± 0.066	0.276 ± 0.062	0.260 (0.143–0.445)	0.278 (0.155–0.482)	0.255 (0.181–0.562)	0.943^a^
WEMT [ms]	21.742 (20.585–23.230)	21.862 (20.536–23.701)	21.454 ± 1.760	21.823 (20.070–23.544)	21.997 ± 0.467	21.879 ± 0.460	<0.001^a^
A1DLAr [mm^2^]	0.180 ± 0.027	0.176 ± 0.023	0.185 ± 0.023	0.190 ± 0.030	0.206 ± 0.038	0.221 ± 0.045	<0.001^b^
HCDLAr [mm^2^]	3.457 ± 0.549	3.577 ± 0.555	3.470 ± 0.590	3.878 (2.433–6.218)	3.981 (2.846–7.195)	4.067 ± 0.520	<0.001^a^
A2DLAr [mm^2^]	0.233 ± 0.041	0.234 (0.171–0.369)	0.232 (0.167–0.373)	0.260 (0.152–0.736)	0.280 (0.152–0.534)	0.307 ± 0.056	<0.001^a^
A1dArcL [mm]	−0.019 (−0.029 to −0.008)	−0.019 (−0.022 to −0.005)	−0.019 ± 0.003	−0.020 ± 0.004	−0.021 (−0.040 to −0.012)	−0.025 ± 0.007	<0.001^a^
HCdArcL [mm]	−0.135 ± 0.028	−0.127 ± 0.024	−0.115 ± 0.020	−0.119 (-0.230 to −0.014)	−0.115 (−0.231 to −0.019)	−0.115 ± 0.032	<0.001^a^
A2dArcL [mm]	−0.022 (−0.036 to −0.004)	−0.023 (−0.040 to −0.016)	−0.023 (-0.036 to -0.016)	−0.025 (−0.057 to 0.006)	−0.029 ± 0.008	-0.031 (-0.051 to 0.002)	<0.001^a^
dArcLM [mm]	−0.153 ± 0.035	−0.148 ± 0.030	−0.146 ± 0.064	−0.142 (−0.452 to −0.034)	−0.135 (−0.399 to −0.041)	−0.137 (−0.270 to -0.050)	0.023^a^
MIR [mm^−1^]	0.167 ± 0.016	0.178 (0.140–0.259)	0.199 ± 0.024	0.209 ± 0.026	0.223 (0.170–0.336)	0.251 ± 0.040	<0.001^a^
DA Ratio 2	4.188 (3.081–5.946)	4.646 ± 0.440	4.880 ± 0.596	5.409 ± 0.786	6.208 ± 0.861	6.615 ± 1.013	<0.001^a^
PachySlope [μm]	50.435 (29.036–98.805)	50.480 ± 10.247	55.290 ± 13.335	65.865 (−24.281–145.630)	76.817 (38.351–154.705)	98.906 ± 24.373	<0.001^a^
DA Ratio 1	1.558 (1.407–1.729)	1.593 ± 0.044	1.613 ± 0.052	1.650 (1.509–1.918)	1.710 (1.560–1.971)	1.744 ± 0.074	<0.001^a^
ARTh	415.883 ± 86.639	392.170 ± 92.140	322.116 (195.308–603.057)	226.520 (28.989–630.625)	174.476 (71.233–417.461)	133.123 ± 56.762	<0.001^a^
bIOP [mmHg]	14.7 (10.8–26.5)	14.3 ± 1.9	14.6 (10.4–29.2)	13.3 ± 2.3	12.4 ± 2.2	12.6 ± 2.9	<0.001^a^
IR [mm^−1^]	7.918 (5.300–12.555)	8.898 ± 1.025	9.698 ± 1.192	10.858 ± 1.716	11.967 (8.085–17.603)	13.401 ± 2.298	<0.001^a^
SPA1	102.040 ± 19.505	85.088 ± 16.692	77.961 ± 18.804	59.822 ± 15.639	47.491 ± 12.512	41.476 ± 14.298	<0.001^b^
CBI	0.065 (0.000–1.000)	0.404 (0.001–1.000)	0.957 (0.000–1.000)	1.000 (0.001–1.000)	1.000 (0.943–1.000)	1.000 (0.970–1.000)	<0.001^a^

a Non-parametric Kruskal–Wallis test.

b One-way ANOVA test.

**FIGURE 1:**
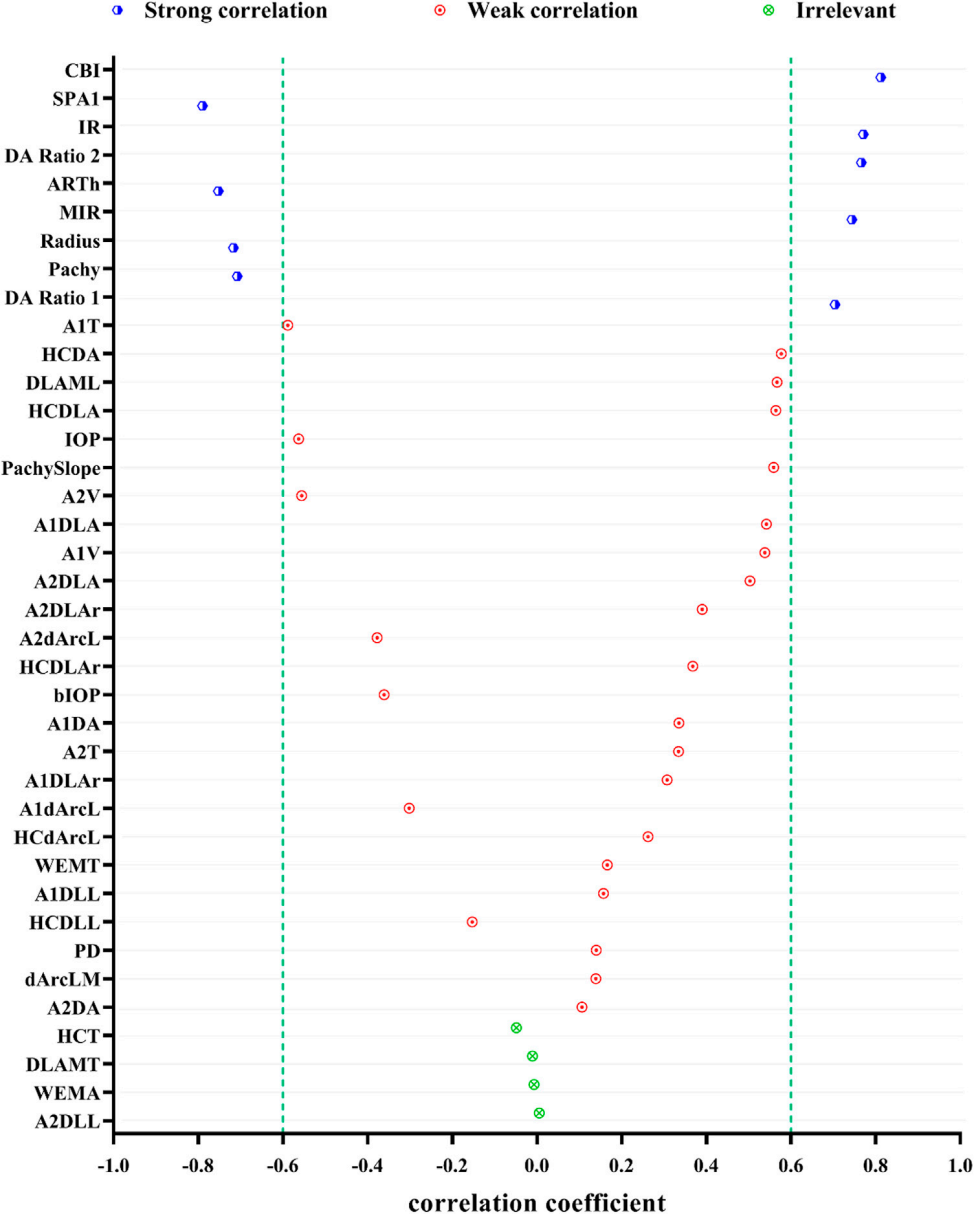
Spearman correlations of DCR parameters and the developmental grades of keratoconus (strong correlation: 0.6 ≤ |*r*| < 1 and *p* < 0.05, weak correlation: |*r*| < 0.6 and *p* < 0.05, irrelevant: *p* ≥ 0.05).

**FIGURE 2:**
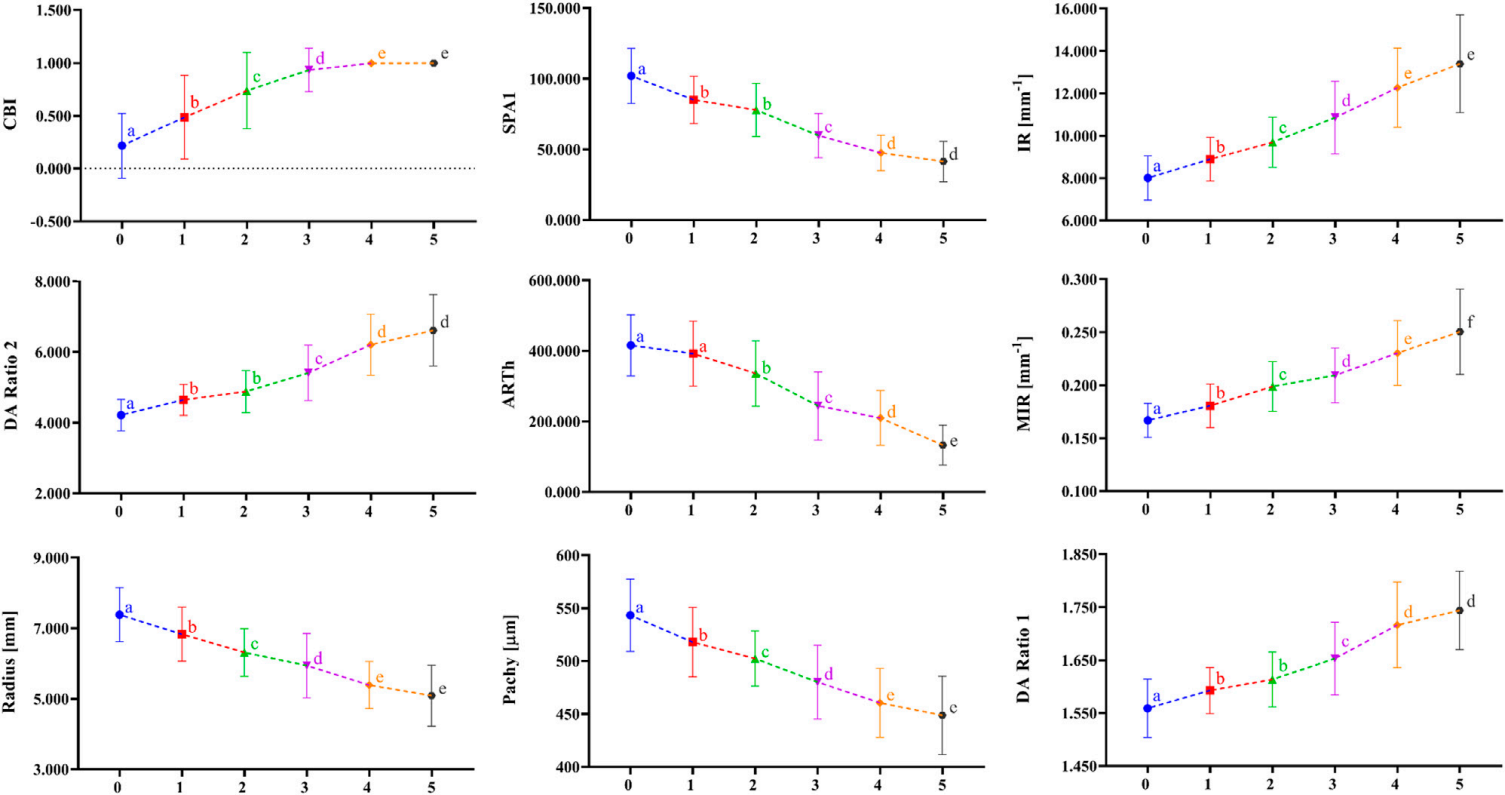
The change tendency of DCR parameters, which are highly correlated as disease progression with the disease stage increasing. a, b, c, d, and e indicate that the parameters in a certain stage are statistically different from the corresponding parameters in the previous adjacent stage (*p* < 0.01); 0 = Normal group, 1 = FFKC group, 2 = CKC Stage I group, 3 = CKC Stage II group, 4 = CKC Stage III group, 5 = CKC Stage IV group.

Figure 1 shows the correlation among 38 parameters measured by Corvis ST and developmental grades of keratoconus. The results unveiled that there were nine DCR parameters that were strongly correlated with Rank-group (|*r*| > 0.6), and they were CBI, SPA1, IR, DA Ratio 2, ARTh, MIR, Radius, and Pachy in the order of correlation from high to low. Figure 2 shows the change trend of the nine DCR parameters screened above with the increase of disease stage. Obviously, these nine parameters would change significantly in the process of disease progression (normal to CKC stage IV), that is, gradually increased or decreased.

**FIGURE 3:**
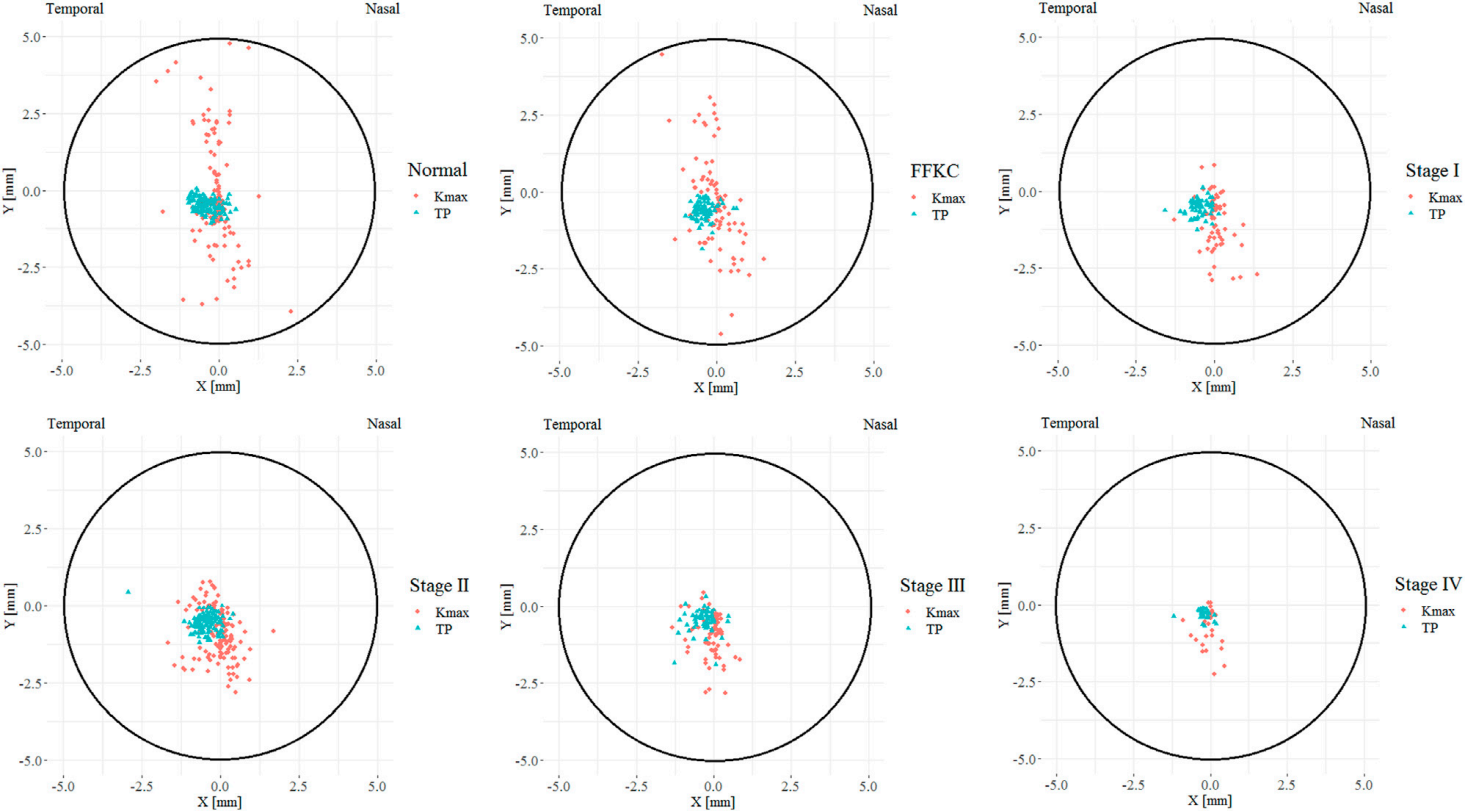
The coordinate distribution of TP and Kmax in different developmental grades of keratoconus, where the X coordinate of the left eye was flipped on the vertical axis to reflect the direction of the right eye (TP = Thinnest point; Kmax = Maximum keratometry of the front surface).

**FIGURE 4:**
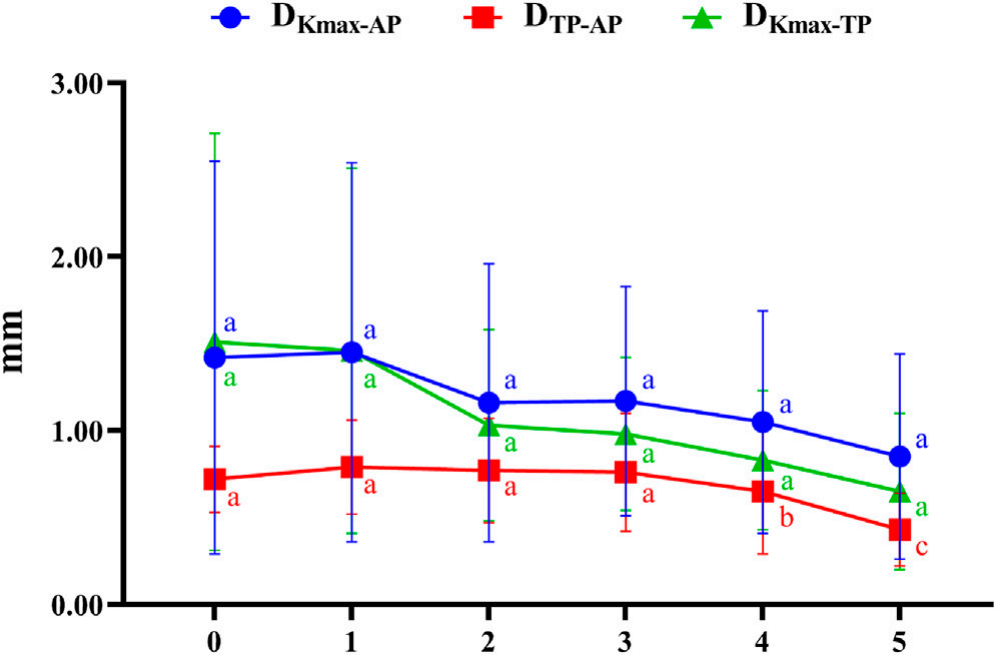
The variation trend of three typical landmark distance parameters with the progress of disease stage (0 = Normal group, 1 = FFKC group, 2 = CKC Stage I group, 3 = CKC Stage II group, 4 = CKC Stage III group, 5 = CKC Stage IV group; D_Kmax-AP_: the absolute distances from the maximum curvature of the anterior surface to the apex of the cornea; D_Kmax-TP_: the absolute distances from the maximum curvature of the anterior surface to the thinnest point; D_TP-AP_: the absolute distances from the cornea apex (geometric center of the examination [x = 0; y = 0]) to the thinnest point).

Figure 3 illustrates the coordinates of Kmax and TP in different developmental grades of keratoconus, and Figure 4 depicts the variation trend of three typical landmark distance parameters of geometric landmarks with the progress of disease stage. As shown in Figure 3, from normal to CKC stage IV, the coordinates of Kmax were gradually concentrated from dispersion, and finally tended to the inferior temporal region, while the coordinates of TP were always concentrated in the inferior temporal region. However, it can be seen from Figure 4 that although D_Kmax-AP_, D_Kmax-TP_, and D_TP-AP_ all showed a gradual decreasing trend with the progress of the disease, the first two (D_Kmax-AP_ and D_Kmax-TP_) did not change significantly, and only D_TP-AP_ significantly approached AP in the later stage of disease development.

**FIGURE 5:**
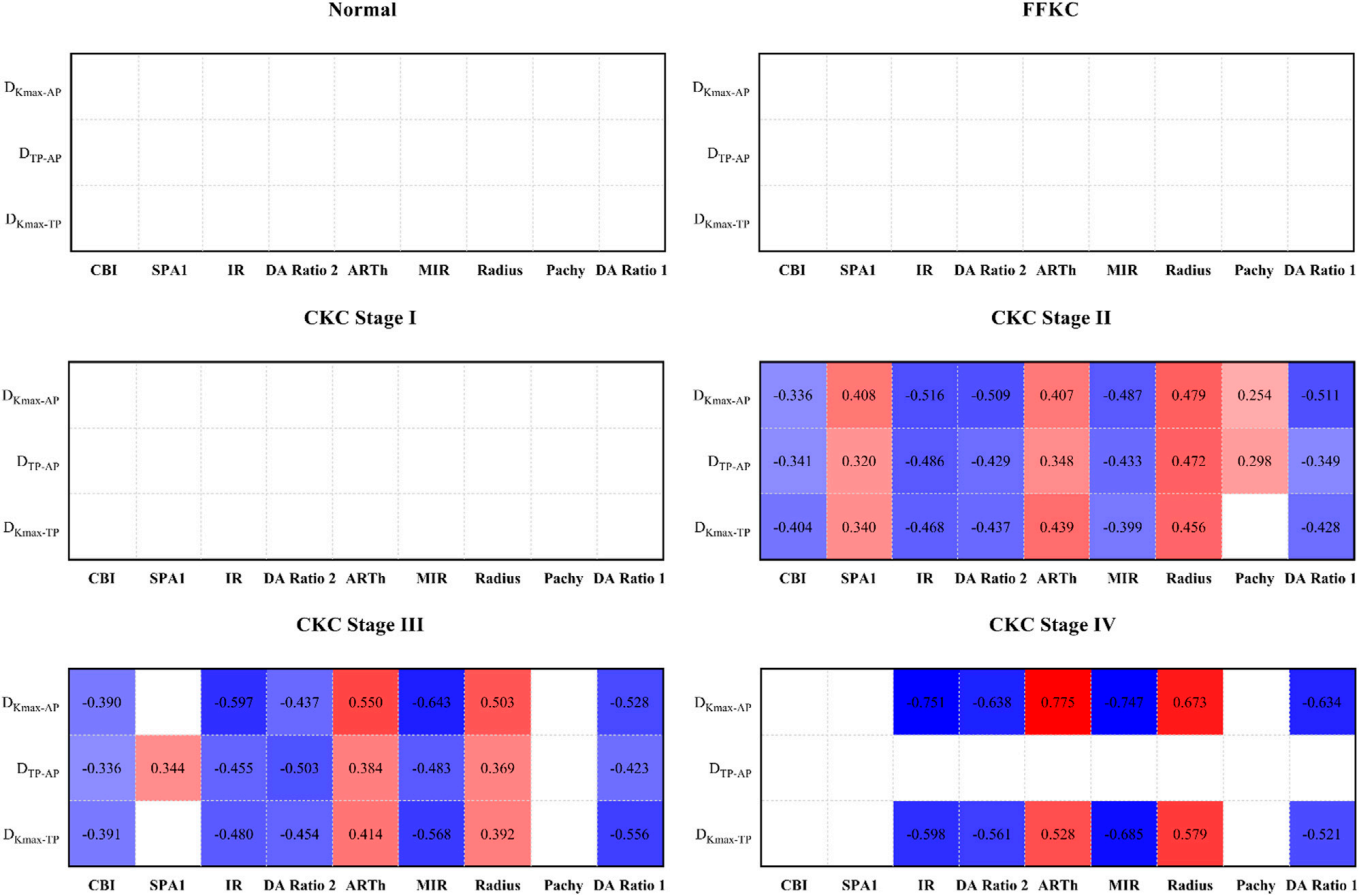
Correlation between the three geometric landmark distance parameters and the nine DCR parameters in each grade from normal to CKC Stage IV (the small lattice filled with color indicates that the correlation is statistically significant, in which red indicates that the correlation coefficient is positive, blue indicates that the correlation coefficient is negative, and the depth of color indicates the absolute value of the correlation coefficient).

Figure 5 represents the correlation between the three geometric landmark distance parameters and the abovementioned nine DCR parameters in each grade from normal to CKC stage IV. From the results, it could be seen that starting from CKC stage II, the distance parameters of the three geometric landmarks were correlated with the DCR parameters (*p* < 0.0056), and with the aggravation of disease, the correlation between the two parameters of D_Kmax-AP_ and D_Kmax-TP_ with the DCR parameters was basically strengthened.

## Discussion

The purpose of the present study was to analyze the changes in coordinates and distances between the three typical geometric landmarks, namely, the thinnest point (TP), the maximum curvature (Kmax), and corneal apex (AP) during the development of keratoconus (Normal, FFKC, and CKC stage I to CKC stage IV), as well as to explore the potential relationship among these changes and the abnormalities of corneal biomechanics.

Our results showed that from normal cornea to CKC stage IV, the coordinates of Kmax gradually tended to focus from the scattered distribution, and finally concentrated on the region under the temporal cornea. However, although the coordinates of Kmax were converging gradually, in fact, its variation range was basically stable within the range of 2 mm in diameter. For TP, as mentioned in other studies (Muftuoglu et al., 2013; Huseynli et al., 2018), it is always concentrated in the inferior temporal region of the cornea, indicating inferior decentration of the thinnest point of the cornea in eyes with early keratoconus. In addition, although there was no significant change in D_Kmax-AP_ and D_Kmax-TP_ in the process of disease progression, it should be pointed out that D_Kmax-TP_ values of each disease grade from FFKC were basically smaller than D_Kmax-AP_, which also illustrated that Kmax might be more inclined to corresponding TP than AP with the development of keratoconus to a certain extent.

Previous studies (Kaya et al., 2007; Ashwin et al., 2009) found that the average distance between the TP and AP in normal eyes was 0.52–1.01 mm, and that in keratoconus eyes was 0.78 mm, which was basically consistent with the results of the current study: normal group (0.72 mm), FFKC group (0.79 mm), and CKC group (0.70 mm). It can be seen from the results of this study that from CKC stage II, the value of D_TP-AP_ would gradually decrease with significant changes, that is, TP would be relatively close to AP in the late stage of disease development. Combined with the discussion in the previous paragraph, it seems that in the late development of keratoconus disease, there will be a trend of proximity or merging between the typical landmark positions of the cornea.

In the present study, there were nine DCR parameters (CBI, SPA1, IR, DA Ratio 2, ARTh, MIR, Radius, Pachy, and DA Ratio 1) that had strong correlation with Rank-group; that is to say, they could well represent the changes in morphological characteristics and biomechanical behavior of cornea during the progress of keratoconus. Studies (Kataria et al., 2019; Yang et al., 2019) pointed out the significant role of these parameters in the diagnosis of keratoconus, which was confirmed by the area under the receiver operating characteristic (ROC) curve. Among the above parameters, in addition to Pachy, ARTh is another DCR parameter that more characterizes the changes of corneal geometric characteristics, which is calculated by the division between corneal thickness at the thinnest point and pachymetric progression index, and a lower value means a faster increase of thickness toward the periphery or a thinner cornea (Vinciguerra et al., 2016). The DA Ratio 1 and DA Ratio 2 are measured at 1 or 2 mm from the center (Wang et al., 2017), and a greater value indicates less resistance to the cornea or a softer cornea. For SPA1, it is developed by using displacement of the apex from the undeformed state to first applanation in the deformation process, and it more characterizes the stiffness behavior of cornea to resist deformation (Vinciguerra et al., 2016). That is, the smaller the SPA1, the smaller the overall stiffness of the cornea. The MIR is the maximum value of radius of curvature during concave phase of the deformation (Yang et al., 2019), while IR is defined as the area under the inverse radius curve with respect to time, and with the progress of keratoconus, both gradually increases, that is, the cornea gradually softens. Of course, the significant trend of the above parameters that can characterize the biomechanical properties of cornea with the increase of disease developmental grades further shows that the cornea will gradually soften during the process of disease progression, that is, the biomechanical properties will gradually weaken (Scarcelli et al., 2014).

According to the results, we found that starting from CKC stage II, the three geometric landmark distance parameters, namely, D_Kmax-TP_, D_Kmax-AP_, and D_TP-AP_, began to be correlated with the selected biomechanical parameters, and with the aggravation of disease, the correlation between the two parameters (D_Kmax-TP_ and D_Kmax-AP_) and the corneal biomechanical parameters was basically strengthened. On this basis, if we synthesize the above discussion, that is, there will be certain proximity or merger between the typical landmark positions of cornea in the later stage of disease development, it is not difficult to believe that the weakening of corneal biomechanical properties may be accompanied by the merger between the typical landmark positions of cornea, especially in the later stage of disease progression.

Of course, our study also had certain limitations. First of all, we did not consider the cone center and the distances in this study because the cone centers of keratoconus were not available in the Pentacam data. Thus, if we can obtain the cone centers of anterior and posterior corneal surfaces by using the Pentacam data, further relevant analysis on the coordinate of the cone center should be carried out in the future research. In addition, this study was not a longitudinal study in the strict sense. The reason why the cross-sectional data were used to study some changes in the development of keratoconus was that the course of keratoconus disease itself was irreversible and would continue to deteriorate over time.

In conclusion, in the later stage of keratoconus, the relationship between the three typical landmark distance parameters and DCR parameters is stronger, and even the weakening of corneal biomechanical properties may be accompanied by the merger of typical landmark positions. It is believed that these findings have certain value for us to further understand the significance of corneal response parameters under external force. Moreover, the coordinate of Kmax may also be used as a reference parameter to judge the disease stage in the future; that is, it will gradually tend to focus from dispersion with the progress of the disease.

## Data Availability

The original contributions presented in the study are included in the article/supplementary material. Further inquiries can be directed to the corresponding authors.

## Glossary

A1DA: Deformation amplitude at the first applanation
A1dArcL: Chang in arc length (length along the curved line) at first applanation
A1DLA: Deflection amplitude at the first applanation
A1DLAr: Deflection area at the first applanation
A1DLL: Deflection length at the first applanation
A1T: Time from the initiation of air puff until the first applanation
A1V: The first velocity of applanation
A2DA: Deformation amplitude at the second applanation
A2dArcL: Chang in arc length (length along the curved line) at second applanation
A2DLA: Deflection amplitude at the second applanation
A2DLAr: Deflection area at the second applanation
A2DLL: Deflection length at the second applanation
A2T: Time from the initiation of air puff until the second applanation
A2V: The second velocity of applanation
ANOVA: One-way analysis of variance
AP: Corneal thickness at the apex
ARTh: Ambrósio relational thickness to the horizontal profile
bIOP: Biomechanical-corrected intraocular pressure
CBI: Corvis Biomechanical Index
CH: Corneal hysteresis
CKC: Clinical keratoconus
Corvis ST: Corneal Visualization Scheimpflug Technology
CRF: Corneal resistance factor
DA Ratio 1: The maximal value of the ratio between deformation amplitude at the apex and that at 1 mm from the corneal apex
DA Ratio 2: The maximal value of the ratio between deformation amplitude at the apex and that at 2 mm from the corneal apex
dArcLM: Delta arc length max
DCR: Dynamic corneal response
D_Kmax-AP_: The absolute distances from the maximum curvature of the anterior surface to the apex of the cornea
D_**Kmax-TP**_: The absolute distances from the maximum curvature of the anterior surface to the thinnest point
DLAML: Max length at deflection amplitude
DLAMT: Max time at deflection amplitude
D_**TP-AP**_: The absolute distances from the cornea apex to the thinnest point
FFKC: Forme fruste keratoconus
HCDA: Deformation amplitude at the highest concavity
HCdArcL: Chang in arc length (length along the curved line) at the highest concavity
HCDLAr: Deflection area at the highest concavity
HCDLL: Deflection length at the highest concavity
HCT: Time from the start until the highest concavity
IOP: Intraocular pressure
IR: Integrated radius
Kmax: Maximum keratometry from the anterior corneal surface
Kmax X: The position of maximum keratometry in X direction relative to apex
Kmax Y: The position of maximum keratometry in Y direction relative to apex
MIR: Max inverse radius
ORA: Ocular Response Analyzer
Pachy: Pachymetry
PD: Peak distance
QS: Quality specification
Radius: Radius of curvature
SD: Standard deviation
SPA1: Stiffness parameter at first applanation
TKC: Topographic keratoconus classification
TP: Corneal thickness at the thinnest point
TP X: The X position of thinnest corneal thickness related to apex
TP Y: The Y position of thinnest corneal thickness related to apex
WEMA: Max amplitude of whole eye movement
WEMT: Max time of whole eye movement
