# Corrigendum: Distribution of corneal geometric landmarks and relationship between their distances and biomechanical parameters in the development of keratoconus

**DOI:** 10.3389/fbioe.2022.1104956

**Published:** 2023-01-10

**Authors:** Lei Tian, Hui Zhang, Li-Li Guo, Xiao Qin, Di Zhang, Lin Li, Ying Wu, Ying Jie, Haixia Zhang

**Affiliations:** ^1^ Beijing Institute of Ophthalmology, Beijing Tongren Eye Center, Beijing Tongren Hospital, Capital Medical University and Beijing Ophthalmology and Visual Sciences Key Laboratory, Beijing, China; ^2^ Beijing Advanced Innovation Center for Big Data-Based Precision Medicine, Beihang University & Capital Medical University, Beijing, China; ^3^ School of Biomedical Engineering, Capital Medical University, Beijing, China; ^4^ Department of Medical Engineering, Peking Union Medical College Hospital, Chinese Academy of Medical Sciences, Beijing, China; ^5^ The First People’s Hospital of Xuzhou, Jiangsu, China; ^6^ Beijing Key Laboratory of Fundamental Research on Biomechanics in Clinical Application, Capital Medical University, Beijing, China; ^7^ Department of Ophthalmology, Chinese People’s Liberation Army General Hospital, Beijing, China

**Keywords:** keratoconus, forme fruste keratoconus, morphology, biomechanics, geometric landmark

In the published article, there was an error in affiliations 5 and 6. Instead of “Department of Ophthalmology, Chinese People’s Liberation Army General Hospital, Beijing, China,” affiliation 5 should be “The First People’s Hospital of Xuzhou, Jiangsu, China.” Instead of “The First People’s Hospital of Xuzhou, Jiangsu, China,” affiliation 6 should be “Beijing Key Laboratory of Fundamental Research on Biomechanics in Clinical Application, Capital Medical University, Beijing, China.”

The authors apologize for this error and state that this does not change the scientific conclusions of the article in any way. The original article has been updated.

